# Three Case Reports of Rhupus Syndrome: An Overlap Syndrome of Rheumatoid Arthritis and Systemic Lupus Erythematosus

**DOI:** 10.1155/2018/6194738

**Published:** 2018-01-21

**Authors:** Gul Devrimsel, Munevver Serdaroglu Beyazal

**Affiliations:** Department of Physical Medicine and Rehabilitation, Faculty of Medicine, Recep Tayyip Erdogan University, Rize, Turkey

## Abstract

We present the clinical and serological characteristics of three patients with rhupus. The 3 patients with rhupus presented ACR criteria for SLE as well as for RA, ANA positive with a titer of 1/100 in all patients, and positive anti-DNA in 2 of the 3 patients, with the predominance of symmetrical polyarthritis. We found anti-CCP positivity and rheumatoid factor positivity and high titers in all patients, positive anti- anti-SSA in one patient, and positive anti- anti-Sm in one patient. Renal and liver function tests were normal in all patients. The 3 patients achieved clinical remission with DMARD treatment.

## 1. Introduction

Systemic lupus erythematosus (SLE) and rheumatoid arthritis (RA) are autoimmune diseases with different clinical and serological features affecting various organs and systems. Although SLE can affect the skin, lungs, and kidneys, it can affect the joints (90%). Rheumatoid arthritis has a worldwide prevalence of approximately in 0.5–1.0% of adult population and SLE prevalent in 6.5–27.7/100,000 [1, 2]. “Rhupus” is a term used to describe patients with coexistence of RA and SLE [3]. It is discussed that the question of whether RA and SLE occur in the same patient, the so-called “rhupus,” or whether any deforming and erosive disease might be integral to the arthritis of SLE. There is evidence to support the presence of rhupus as a true overlap syndrome [4, 5]. Rhupus syndrome is a rare clinical entity which has an estimated prevalence rate of 0.09% [3]. We present a series of three cases of patients with a diagnosis of rhupus.

## 2. Case Reports

### 2.1. Patient 1

**Figure 1:**
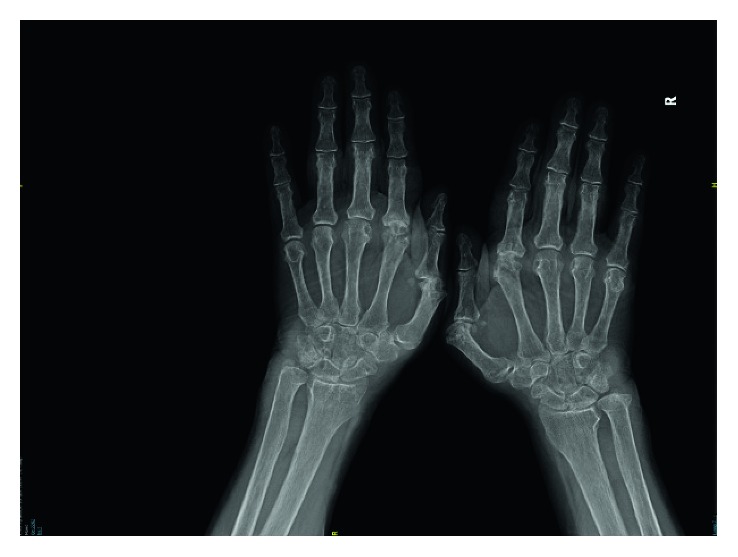
X-ray findings of the hands (patient 1).

A 50-year-old female was admitted with the complaints of intermittent pain in hand joints and morning stiffness for the past nine years. She gave a history of alopecia, fever, fatigue, malar rash, and oral ulcers for the last nine years. On physical examination, she had oral cold sores, tenderness and swelling on the 2. and 3. proximal interphalangeal joints bilaterally, tenderness on the wrist joints bilaterally, swan neck deformity on second finger of the right hand, and bilateral ulnar deviation. X-ray findings of the hands are shown in Figure 1.

Laboratory studies showed normal blood count, an erythrocyte sedimentation rate of 45 mm/h, C-reactive protein of 3.39 mg/dL, rheumatoid factor of 393 IU/mL, anti-CCP level of 500 U/mL, antinuclear antibodies (ANA) 1/100 positive, anti-dsDNA positive with 43.2 positive, and C4 13.2 mg/dl. Urinalysis showed microscopic hematuria (red blood cells 9/high-power field). Urine protein-to-creatinine ratio (UP/CR) is 50 mg protein per gram creatinine (normal < 30 mg protein for 24 hours in urine). Renal and liver function tests were normal.

### 2.2. Patient 2

**Figure 2:**
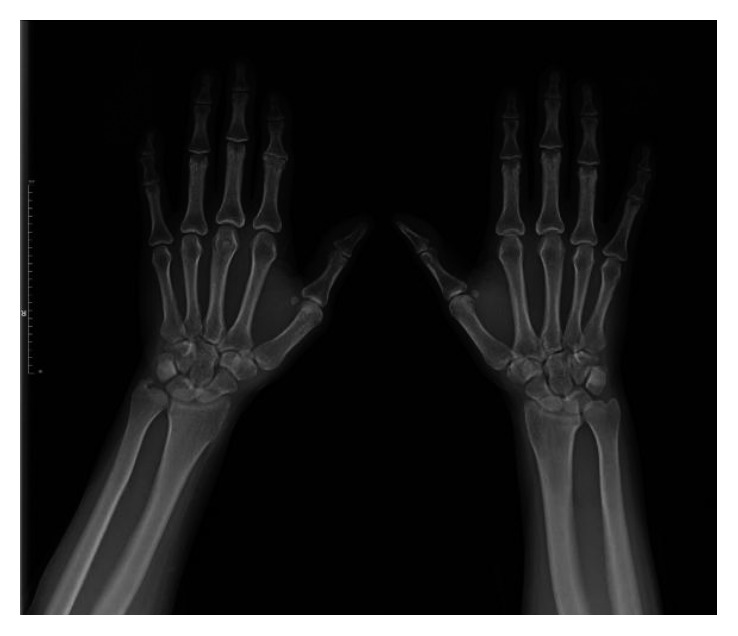
X-ray findings of the hands (patient 2).

A 26-year-old female was referred to our department with the complaint of bilateral hand joint pain for the last eighteen months. She gave a history of alopecia, Raynaud phenomenon, erythema nodosum, fatigue, malar rash, and oral ulcers. On physical examination, she had bilateral 1. proximal interphalangeal joint tenderness, right knee joint swelling, and right wrist joint tenderness. X-ray findings of the hands are shown in Figure 2.

Laboratory studies showed normal blood count, an erythrocyte sedimentation rate of 54 mm/h, C-reactive protein of 2.40 mg/dL, rheumatoid factor of 78.2 IU/mL, anti-CCP level of 47.9 U/mL, ANA 1/100, anti-dsDNA negative, and anti-Sm positive. Urinalysis showed microscopic hematuria (red blood cells 6/high power field). Urine protein-to-creatinine ratio (UP/CR) is 35 mg protein per gram creatinine (normal < 30 mg protein for 24 hours in urine).

Renal and liver function tests were normal.

### 2.3. Patient 3

**Figure 3:**
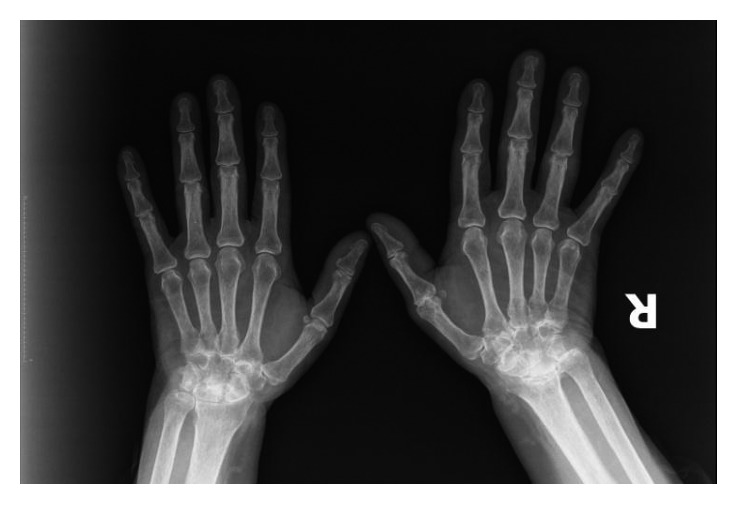
X-ray findings of the hands (patient 3).

A 40-year-old female presented with the complaint of intermittent joint pain for the past six years. For the past 2 years, she also had oral ulcers and malar rash. On physical examination, she had right 4. proximal interphalangeal joint swelling and tenderness, left 2. metacarpophalangeal tenderness, bilateral wrist joint tenderness, malar rash, oral ulcers, and morning stiffness. X-ray findings of the hands are shown in Figure 3.

Laboratory tests revealed a normal blood count, an erythrocyte sedimentation rate of 56 mm/h, C-reactive protein of 2.36 mg/dL, rheumatoid factor of 84.1 IU/mL, and anti-CCP level of 63.2 U/mL. The ANA (1/100), anti-dsDNA, and anti-SSA were positive, but anti-Sm was negative. Urinalysis showed microscopic hematuria (red blood cells 7/high-power field). Urine protein-to-creatinine ratio (UP/CR) is 40 mg protein per gram creatinine (normal < 30 mg protein for 24 hours in urine). Renal and liver function tests were normal.

## 3. Diagnosis and Treatment

Rhupus syndrome was diagnosed in three patients, and an oral treatment with methotrexate (15 mg/week), folic acid (5 mg/week), and hydroxychloroquine (200 mg/twice a day) were begun. Patient 1 achieved clinical remission within the second month, and the other 2 patients achieved clinical remission within the first month.

## 4. Discussion

Rhupus is a complicated condition, and a debate still exists with regard to its definition and diagnosis. Amezcua-Guerra et al. [6] stated that the presence of anti-CCP antibody, high C-reactive protein values, and shared epitope are characteristics of rhupus. Previous reports have demonstrated that anti-double-stranded DNA (anti-dsDNA), anti-Sm (both highly specific for SLE), anti-SSA, anti-SSB, anti-ribonucleoprotein, ANA, anti-cardiolipins, and rheumatoid factor are positive in patients with rhupus [3, 7]. There is a shared autoimmunity in the pathogenesis of RA and SLE. Genetic studies supported this shared autoimmunity [6]. It was identified that TAP2^∗^0201(RA and SLE) and TNF-308A gene variants in the same chromosomal region increase susceptibility to autoimmune diseases such as RA, SLE, and Sjögren syndrome [8]. On the other hand, PDCD1, STAT4, FCRL3, and PTPN22 genes were found to be associated with RA and SLE. It is demonstrated that HLA-DR1 and HLA-DR2 alleles were significantly increased in patients with rhupus [7, 9–11]. Chan and colleagues suggested that anti-CCP+ patients with SLE are more likely to have erosive arthritis, and anti-citrullinated antibodies may have a pathogenic role in the development of major erosions [12]. Amezcua-Guerra et al. demonstrated that the anti-CCP antibody frequency and titers in patients with rhupus were similar to those in patients with RA but significantly higher than those in patients with nonerosive arthropathy in SLE [6]. Rhupus syndrome is characterized by symmetric polyarthritis of the small and large joints and symptoms of SLE and by the presence of specific autoantibodies with high specificity (anti-dsDNA antibody or anti-Smith for SLE and rheumatoid factor or anti-citrullinated peptide antibodies for RA) in our patients.

Consequently, rhupus arthropathy is an overlapping syndrome of rheumatoid arthritis and systemic lupus erythematosus that is defined by erosive polyarthritis accompanied by an overlap of clinical and immunological symptoms. Physicians should remain alert to manifestations of autoimmunity and overlapping disease features. We wanted to draw attention to awareness for early recognition and prompt diagnosis in order for the effective treatment of this disease.
